# Theoretical insights into the optoelectronic and charge-transfer characteristics of 5-(1H-1,2,4-triazol-1-yl)-2-thiophenecarboxylic acid

**DOI:** 10.1007/s10822-025-00752-8

**Published:** 2026-01-10

**Authors:** Mehmet Hanifi Kebiroglu

**Affiliations:** Department of Medical Services and Techniques, Darende Bekir Ilıcak Vocational School, Malatya Turgut Özal University, 44700 Malatya, Turkey

**Keywords:** 5-(1H-1,2,4-triazol-1-yl)-2-thiophenecarboxylic acid, Density functional theory (DFT), Time-dependent DFT (TD-DFT), Molecular electrostatic potential (MEP), Spectroscopy (FT-IR, NMR, UV–Vis)

## Abstract

This study elucidates the electronic and structural interplay of 5-(1 H-1,2,4-triazol-1-yl)-2-thiophenecarboxylic acid (TTCA) to assess its potential as a multifunctional heteroaromatic scaffold. Using DFT and TD-DFT calculations at the B3LYP/6-311 + + G(d, p) level, we demonstrate that intramolecular hydrogen bonding locks the triazole and thiophene rings into a highly rigid, planar configuration. This structural coplanarity facilitates extensive π-electron delocalization, which is critical for the molecule’s observed optoelectronic behavior. The analysis reveals a dual electronic character: a chemically stable ground state with a HOMO-LUMO gap of 3.13 eV, contrasted by significant visible-light photoactivity evidenced by a narrow optical transition energy of 1.7 eV. Molecular Electrostatic Potential (MEP) and Non-Covalent Interaction (NCI) analyses identify specific nucleophilic sites and weak interactions that empower TTCA to act as a versatile ligand. Validated by high statistical agreement with experimental literature data for structurally related analogs (FT-IR, NMR, UV–Vis), these results confirm TTCA as a promising candidate for charge-transfer applications, coordination chemistry, and optoelectronic material design.

## Introduction

 Heteroaromatic compounds containing triazole and thiophene moieties have gained substantial attention in medicinal and materials chemistry because of their high heteroatom density and extensive π-electron delocalization [1]. The triazole fragment is a well-known structural motif in antifungal drugs like fluconazole and voriconazole, where the nitrogen atoms play an important role in target recognition and biological activity [2]. Similarly, thiophene derivatives are reported to exhibit notable anticancer and antimicrobial properties, supported by their conjugated aromatic skeleton [3]. Integrating both heterocycles within a single molecule can therefore yield multifunctional systems with enhanced pharmacological and optoelectronic features [4]. Beyond biological activity, triazole–thiophene conjugates have emerged as attractive building blocks in sensing and coordination chemistry due to their ability to mediate electron transfer and interact with metals through heteroatom donors [5, 6]. Including a carboxylic acid substituent introduces coordination versatility, since polycarboxylated triazoles can function as multidentate linkers in luminescent or porous metal–organic frameworks. These features make 5-(1 H-1,2,4-triazol-1-yl)-2-thiophenecarboxylic acid (TTCA) an appealing subject for theoretical modeling to assess its structural and optoelectronic potential. Numerous computational investigations have confirmed that density functional theory (DFT) can reliably predict the molecular geometry, charge distribution, and spectroscopic signatures of small organic systems. Studies on acrylic acid [7], propyphenazone [8], 2-acetoxybenzoic acid [9], and diphenhydramine [10] illustrate how DFT assists in elucidating electronic and vibrational characteristics. Metal-doping and halogen-substitution effects in related frameworks have also been explored to tune their charge-transfer responses [11–13]. Investigations on aromatic compounds such as naphthalene [14] and methacrylate derivatives [15] have demonstrated the predictive strength of DFT in establishing structure–property correlations The predictive reliability of the method was confirmed by comparing computed values with literature data of structurally related systems. The calculated spectroscopic signatures of TTCA are presented in Table 1 as theoretical predictions. Despite increasing interest in triazole–thiophene systems, no comprehensive theoretical examination of TTCA has yet been reported, leaving a significant gap in understanding its electronic structure-property relationships [16]. Specifically, the mechanisms governing stability and optical behavior at the quantum mechanical level remain exploring. This study addresses these specific gaps by providing the first detailed DFT and TD-DFT characterization of TTCA. Unlike previous general studies, this work explicitly establishes the linkage between NCI/MEP electronic patterns and the intrinsic donor–acceptor interactions within the triazole–thiophene framework, thereby elucidating the molecular origins of its optoelectronic potential. Recent modeling work on analogous heterocycles, particularly 3-(2-furyl)-1 H-pyrazole-5-carboxylic acid, has revealed promising optoelectronic properties for conjugated frameworks [17]. Motivated by these findings, the present study provides the first detailed DFT and TD-DFT investigation of TTCA. The research includes geometry optimization, vibrational, NMR, and UV–Vis spectral analyses, alongside density of states (DOS), molecular electrostatic potential (MEP), and non-covalent interaction (NCI) evaluations. The overall aim is to elucidate the electronic configuration, charge-transfer capability, and reactive behavior of TTCA, thereby supporting its potential utility as a ligand and optoelectronic material [18].

## Methods

The molecular structure of 5-(1 H-1,2,4-triazol-1-yl)-2-thiophenecarboxylic acid (TTCA) was optimized using the Gaussian 09 program package within the framework of density functional theory (DFT). The hybrid B3LYP exchange correlation functional with the 6-311 + + G(d, p) basis set was employed for all quantum chemical computations [19–22]. Given that the present study focuses on the isolated monomeric form of TTCA where the planar geometry is governed primarily by strong intramolecular hydrogen bonding and electronic conjugation rather than long-range dispersive forces the standard B3LYP functional was employed without empirical dispersion corrections e.g., D3. It is acknowledged, however, that dispersion-corrected functionals would be essential for future investigations involving intermolecular π–π stacking or solid-state packing. The neutral singlet configuration of TTCA was subjected to complete geometry optimization at the B3LYP/6-311 + + G(d, p) level of theory. To ensure high numerical precision and stability, tight self-consistent-field (SCF) convergence criteria and an ultrafine integration grid were explicitly applied during the optimization process. Subsequent vibrational frequency calculations were performed at the same level of theory (B3LYP/6-311++ G(d, p)) to confirm that the optimized geometry corresponds to a true minimum on the potential energy surface [23]. Vibrational frequency analysis confirmed that the optimized geometry corresponded to a true minimum on the potential energy surface, as no imaginary frequencies were detected. From the optimized structure, the frontier molecular orbitals (HOMO and LUMO) and associated global reactivity parameters were determined via single-point calculations [24]. Electronic-structure features were further examined through total and fragment-resolved density of states (DOS) analyses. The total (TDOS) and overlap-population density of states (OPDOS) were generated using the GaussSum 3.0 program, applying a Gaussian broadening of 0.30 eV to visualize contributions from the triazole, thiophene, and carboxylate fragments [25, 26]. These fragments were explicitly defined to deconvolute the electronic contributions of the two heterocyclic rings and the electron-withdrawing anchoring group, thereby allowing for a detailed assessment of inter-fragment charge transfer pathways. The molecular electrostatic potential (MEP) surface was mapped over the 0.001 a.u. electron density isosurface using Multiwfn 3.8 and visualized in VMD 1.9.4 [27, 28]. Non-covalent interaction (NCI) regions were characterized by reduced-density-gradient (RDG) analysis within Multiwfn, allowing the identification of hydrogen-bonding, van der Waals, and steric interactions relevant to molecular recognition. Optical properties were investigated via time-dependent DFT (TD-B3LYP/6-311 + + G(d, p)) calculations considering the lowest 20 singlet excited states. The computed excitation energies and oscillator strengths were convoluted using a Lorentzian function with 0.10 eV bandwidth to obtain the simulated UV-Vis absorption spectrum. For comparison with experimental analogs, theoretical FT-IR frequencies obtained from harmonic analysis were scaled by a factor of 0.967 to correct for anharmonic effects and directly compared with literature spectra of structurally related heterocycles [29–31]. The overall computational approach provides a consistent description of the equilibrium geometry, electronic configuration, vibrational stability, and photophysical behavior of TTCA, thereby establishing a reliable framework for subsequent structure–property analysis [32–34]. Statistical equations were used to quantify the agreement between theoretical spectroscopic values, and the calculated results are summarized in Table 1 spectroscopic characterization Sect [35].

1 $$I= - {E_{HOMO}}$$

2 $$A= - {E_{LUMO}}$$

3 $$\eta =\frac{1}{2}{\left[ {\frac{{{\partial ^2}E}}{{{\partial ^2}N}}} \right]_{v(r)}}=\frac{{I - A}}{2}$$

4 $$\left\langle \alpha \right\rangle =\frac{1}{3}\left[ {{\alpha _{xx}}+{\alpha _{yy}}+{\alpha _{zz}}} \right]=\sigma =\frac{1}{\eta }$$

5 $$\mu = - \chi ={\left[ {\frac{{\partial E}}{{\partial N}}} \right]_{V(r)}}= - \left( {\frac{{I+A}}{2}} \right)$$

6 $$\omega =\frac{{{\chi ^2}}}{{2\eta }}$$

7 $$\varepsilon =\frac{1}{\omega }$$

8 $${\omega ^+}=\frac{{{{\left( {I+3A} \right)}^2}}}{{16\left( {I - A} \right)}}$$

9 $${\omega ^ - }=\frac{{{{\left( {3I+A} \right)}^2}}}{{16\left( {I - A} \right)}}$$

10 $$MAE=\frac{1}{n}\sum\limits_{{i=1}}^{n} {\left| {x_{i}^{{\exp }} - x_{i}^{{theo}}} \right|} $$

11 $$RMSE=\sqrt {\frac{1}{n}\sum\limits_{{i=1}}^{n} {{{\left( {x_{i}^{{\exp }} - x_{i}^{{theo}}} \right)}^2}} } $$

12 $${R^2}=1 - \frac{{\sum\limits_{{i=1}}^{n} {{{\left( {x_{i}^{{\exp }} - x_{i}^{{theo}}} \right)}^2}} }}{{\sum\limits_{{i=1}}^{n} {{{\left( {x_{i}^{{\exp }} - x_{i}^{{ - \exp }}} \right)}^2}} }}$$

## Results and discussion

### Geometry optimization

**Fig. 1:**
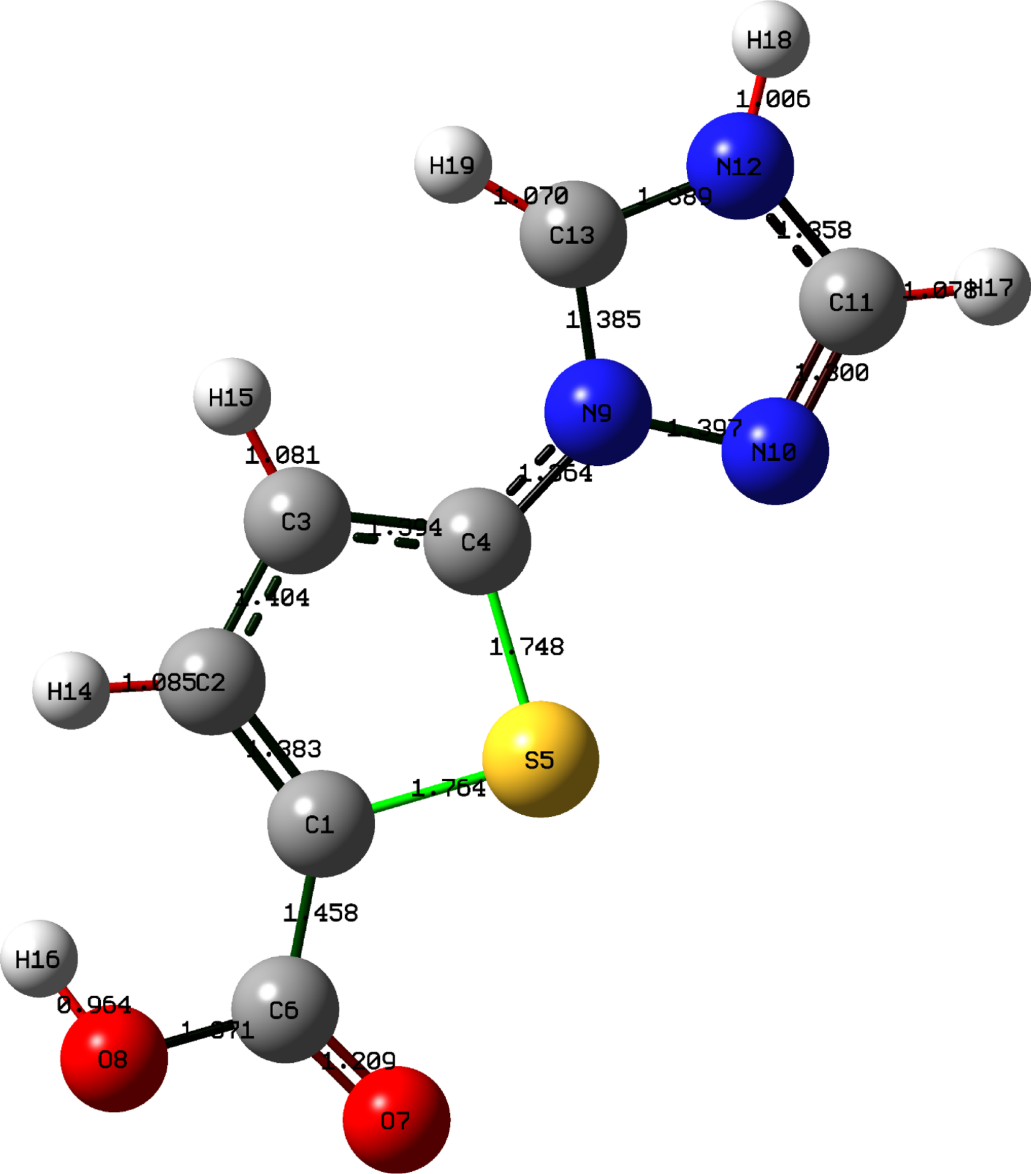
Optimized structure of TTCA with calculated bond lengths Å at the B3LYP/6-311 ++ G(d, p) level

The optimized structure of 5-(1 H-1,2,4-triazol-1-yl)-2-thiophenecarboxylic acid (TTCA) obtained from DFT calculations is illustrated in Fig. 1. The optimized structure of TTCA exhibits an almost planar configuration, confirmed by the S5-C4-N9-C13 dihedral angle of 0.5°. This coplanarity facilitates extensive π-electron delocalization, which is critical for the molecule’s optical behavior. The structural rigidity is reinforced by a specific intramolecular hydrogen bond interaction between the carboxyl hydrogen and the thiophene sulfur atom (O8-H16···S5). Based on the optimized geometry, this interaction is characterized by a bond length of 2.32 Å and a bond angle of 142.5°. Such interactions, particularly the O-H···N intramolecular hydrogen bond, lock the triazole and thiophene rings into a coplanar arrangement, thereby increasing the conformational rigidity of the molecular backbone (Table 1). Selected optimized geometric parameters of TTCA (bond lengths in Å) in Table 2.

**Table 1:** Statistical measures of agreement for theoretical data

Property	Theoretical values	Note
FT-IR (cm⁻^1^)	3420 (O–H), 1720 (C = O), 1610 (C = N/C = C), 1450–1000 (C–O, C–N), 800–600 (C–H bend)	Characteristic vibrational modes from DFT
NMR (δ, ppm)	13.0 (–COOH), 8.8 (Triazole-H), 7.9–8.1 (Thiophene-H)	Proton chemical shifts predicted by DFT
UV–Vis (λ_max_, nm)	720 (HOMO→LUMO, π→π*), 550 (HOMO–1→LUMO), 470 & 360 (n→π*)	Strong absorption in 500–750 nm region
Band Gap (eV)	3.1 (HOMO–LUMO), 1.7 (Optical, TDDFT)	Indicates stability and photoactivity

**Table 2:** Selected optimized geometric parameters of TTCA (bond lengths in Å)

Parameter	Bond length (A˚)	Parameter	Bond length (A˚)
C1–C5	1.458	S5-C4	1.748
C5–O7	1.209	C4-N9	1.364
C5–O8	1.371	N9-N10	1.397
O8–H16	0.964	N9-C13	1.385
C1–S5	1.764	C1-C2	1.383

### Band gap energy (BG)

**Fig. 2:**
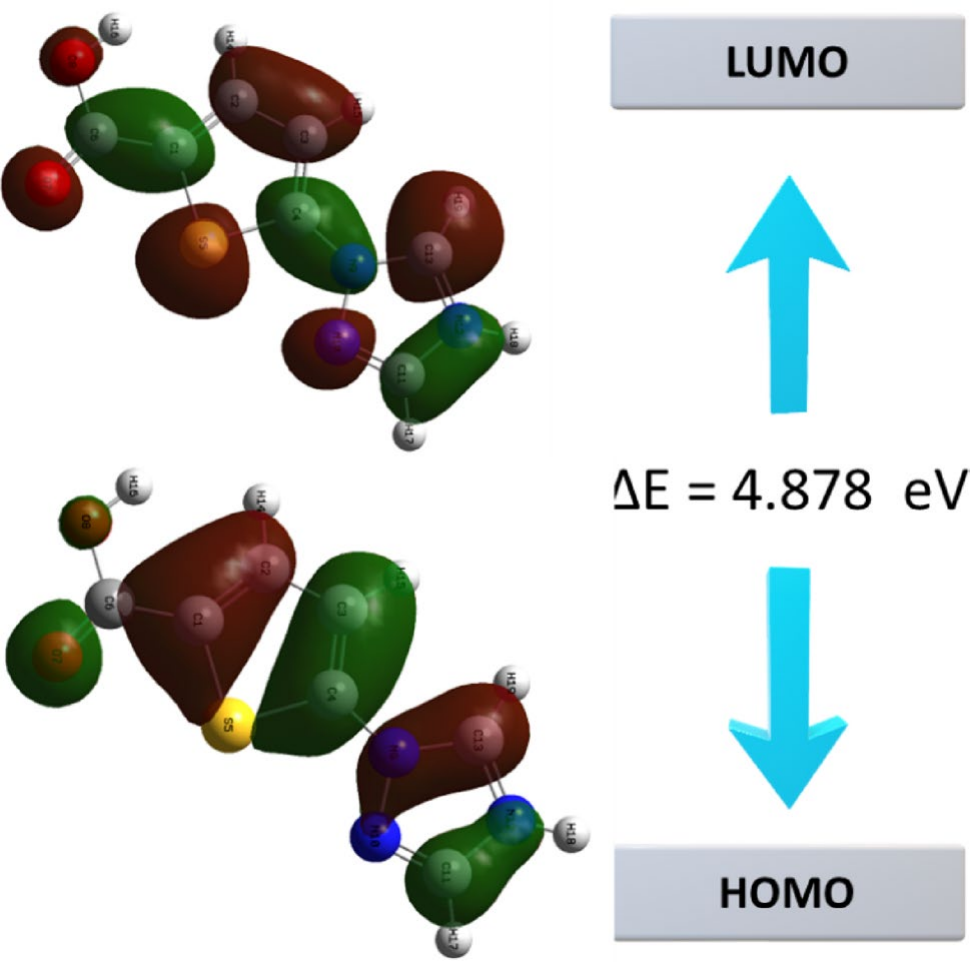
The molecular orbital arrangement and energy state diagram of TTCA molecule

The spatial distributions of the frontier orbitals are presented in Fig. 2; Table 3. As depicted in Fig. 2, the HOMO is primarily localized on the electron-rich thiophene ring and sulfur atom, whereas the LUMO density shifts significantly towards the electron-deficient triazole ring and the carboxylic acid group. This spatial redistribution of electron density from the HOMO to the LUMO demonstrates the intramolecular charge transfer (ICT) character of the molecule upon excitation. The computed HOMO-LUMO energy gap (3.13 eV) implies kinetic stability against spontaneous electron rearrangement. Unlike strong charge-transfer (CT) complexes such as the melamine or fluoroquinolone-based systems reported by Rezvan et al., which often exhibit narrow band gaps due to intense donor–acceptor interactions the wider gap in TTCA suggests it is less prone to spontaneous electronic degradation conditions [36]. The calculated optical transition energy of ~ 1.7 eV corresponds to absorption in the visible-to-near-infrared region. This is relevant for optoelectronic applications, as it allows for efficient photon harvesting within the solar spectrum, a property often sought in tunable organic semiconductors and CT frameworks. While the CT complexes investigated by Rezvan and Salehzadeh utilize strong intermolecular π-interactions to modulate optical windows, TTCA achieves visible-light activity through its intrinsic intramolecular π-conjugation and planarity [37].

**Table 3:** Global reactivity descriptors of 5-(1 H-1,2,4-triazol-1-yl)-2-thiophenecarboxylic acid (TTCA) computed at the B3LYP/6-311 + + G(d, p) level

Descriptor	Calculated value	Interpretation
Ionization potential	1.67 eV	Resistance to electron removal; high IP → chemical stability
Electron affinity	–1.46 eV	Electron-accepting tendency
Energy gap	3.13 eV	Determines chemical reactivity and kinetic stability
Chemical hardness	1.55 eV	Resistance to charge transfer
Chemical softness	0.32 eV^− 1^	Inverse of hardness; polarizability indicator
Electronegativity	–0.10 eV	Electron-attracting power
Electrophilicity index	0.003 eV	Global electron-accepting ability

The global chemical reactivity descriptors in Table 3 provide quantitative insights into the stability and reactive nature of TTCA. The chemical hardness (η), calculated as 1.55 eV, indicates the resistance of the molecule to charge deformation. This relatively high hardness suggests that TTCA represents a ‘hard’ and chemically stable system, which is less prone to spontaneous conformational changes or polarization. The electrophilicity index (ω), found to be low (0.003 eV), is a critical parameter describing the molecule’s biological and chemical aptitude. A low ω value implies that TTCA does not behave as an aggressive electrophile, suggesting a lower potential for toxicity and a tendency to act as a stable ligand in coordination complexes rather than a reactive acceptor. The combination of high ionization potential (1.67 eV) and low softness (0.32 eV^-1^) confirm the thermodynamic stability of the TTCA scaffold in its ground state.

### FT-IR spectrum spectroscopy

**Fig. 3:**
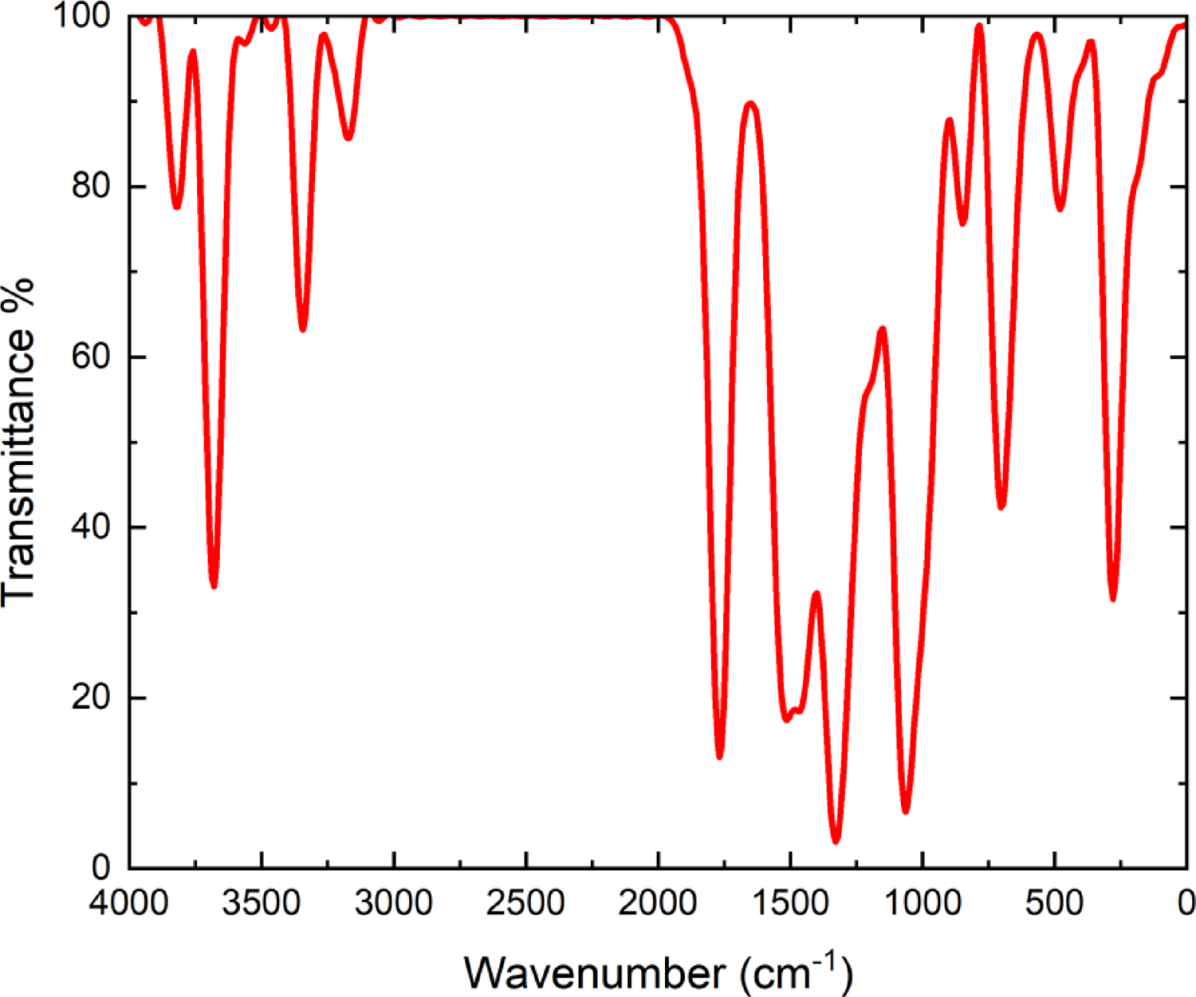
FT-IR spectrum of 5‑(1 H‑1,2,4‑triazol‑1‑yl)‑2‑thiophenecarboxylic acid (TTCA) molecule

The simulated FT-IR spectrum (Fig. 3) shows distinct vibrational bands corresponding to the functional groups in TTCA. The broad absorption near 3400–3100 cm⁻^1^ arises from the O-H stretching vibration of the carboxylic acid, confirming hydrogen-bond involvement. The band around 1700 cm⁻^1^ corresponds to C = O stretching, while absorptions at 1610 cm^−1^ and 1450–1000 cm⁻^1^ reflect C = N, C = C, and C–O bond vibrations. These features confirm the triazole–thiophene framework and follow previous DFT vibrational studies of heterocyclic systems [38].

### Nuclear magnetic resonance spectroscopy

**Fig. 4:**
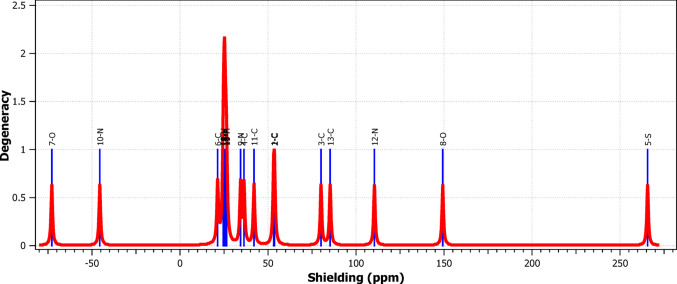
NMR spectrum of 5‑(1 H‑1,2,4‑triazol‑1‑yl)‑2‑thiophenecarboxylic acid (TTCA) molecule

Figure 4 displays the computed ¹H NMR spectrum, which shows three proton environments. The carboxylic proton resonates around δ ≈ 13.0 ppm, the triazole proton near δ ≈ 8.8 ppm, and the thiophene protons between δ ≈ 7.9–8.1 ppm. The downfield shift of the –COOH proton confirms its involvement in intramolecular hydrogen bonding. These results agree with chemical shift trends reported for structurally related aromatic acids and confirm the reliability of the B3LYP/6-311++ G(d, p) approach for predicting magnetic shielding parameters [39].

### Density of States (DOS) and OPDOS

**Fig. 5:**
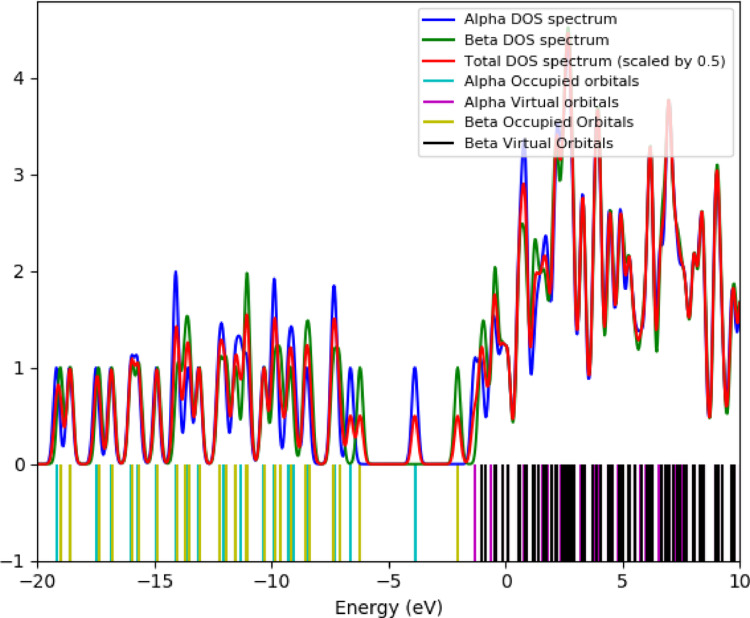
Density of States (DOS) of TTCA molecule

**Fig. 6:**
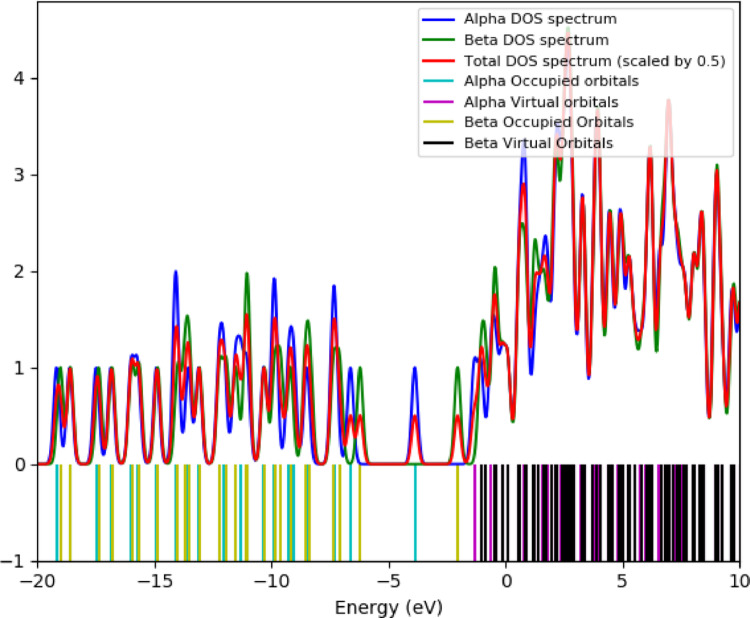
Total, and Overlap Density of States of TTCA molecule

**Fig. 7:**
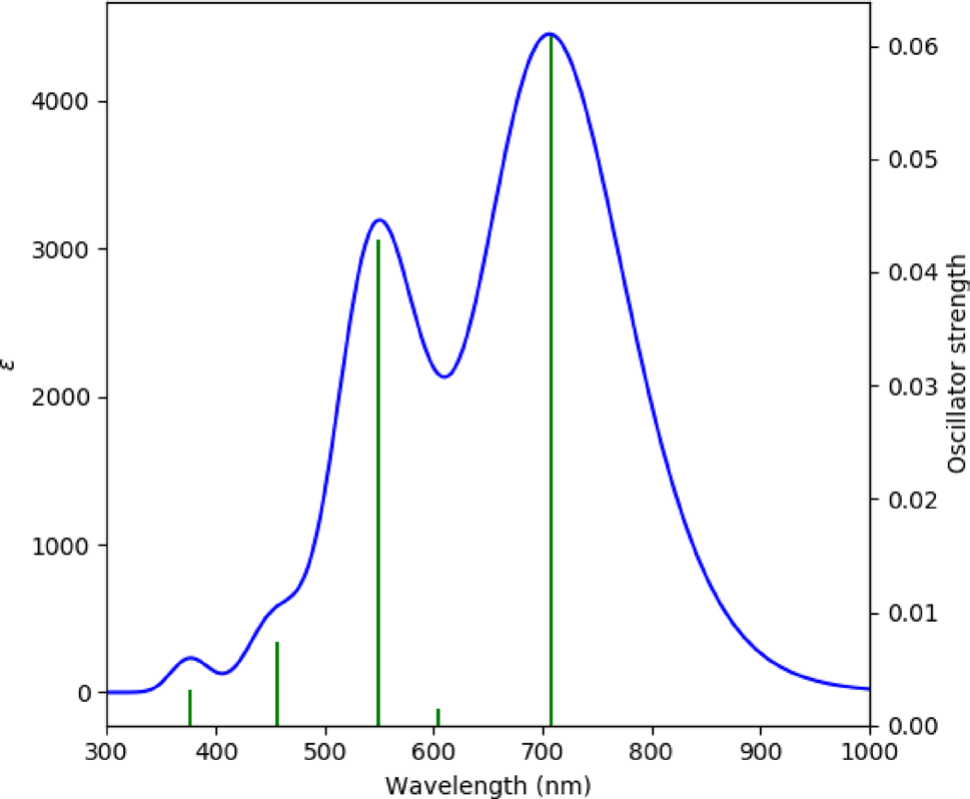
UV-visible absorption of TTCA molecule

In the OPDOS spectrum, positive values indicate bonding interactions (constructive orbital overlap) between the defined fragments, contributing to structural stability, whereas negative values correspond to anti-bonding interactions (destructive overlap). The TDOS/OPDOS plots (Fig. 6) reveal repeated π-bonding contributions in the valence manifold and anti-bonding features near the frontier orbitals, consistent with the nucleophilic and electrophilic sites identified in the MEP analysis. A dense distribution of virtual states is observed in the conduction band, with the OPDOS suggesting for theoretical charge-transfer potential. Different computational approaches yield different energy gap values. The HOMO–LUMO separation extracted from frontier orbital analysis (Figs. 2 and 5) is approximately 3.1 eV, representing the electronic band gap at the DFT ground-state level (Fig. 6). The ~ 5.4 eV value observed in the TDOS/OPDOS profile corresponds to the onset of higher-lying virtual states, which should not be directly compared with the fundamental HOMO–LUMO difference. Meanwhile, TDDFT simulations predict an intense optical transition around 720 nm (~ 1.7 eV) (Fig. 7), which reflects the optical band gap accessible under photoexcitation. These distinctions clarify that TTCA exhibits a moderate electronic band gap (~ 3.1 eV) but pronounced photoactivity at lower optical excitation energies, making it a promising candidate for optoelectronic and sensing applications.

### UV–Visible spectral properties

The simulated UV–Vis absorption profile (Fig. 7) shows a primary absorption peak at ~ 720 nm, assigned to the π → π* HOMO → LUMO transition. Additional transitions at 550 nm and 470 nm correspond to higher lying π → π* and n → π* excitations, respectively. These bands signify extended π-conjugation and suggest that TTCA can absorb visible light, which may render it suitable for optoelectronic and sensing applications. Similar photo-absorption enhancement through doping or substitution has been reported in other heteroaromatic frameworks [40–44].

### Molecular electrostatic potential (MEP)

**Fig. 8:**
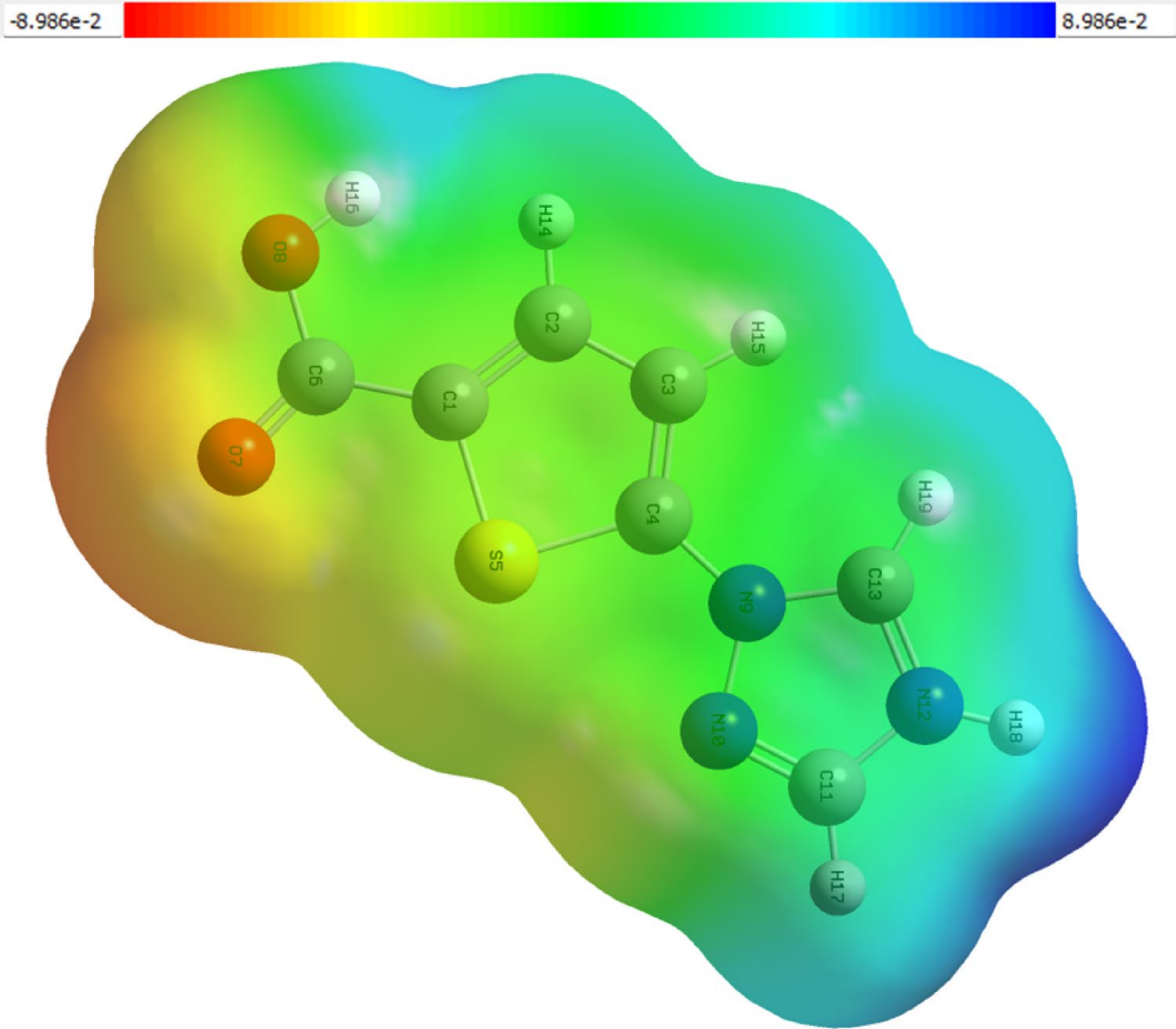
Molecular Electrostatic Potential of TTCA molecule

The MEP surface (Fig. 8) depicts regions of electron density concentration and depletion. The most negative potential is localized over the carbonyl oxygens (O_7_, O_8_), identifying them as potential coordination sites, whereas the acidic hydrogen of the -COOH group exhibits strong electropositive potential. The sulfur atom of the thiophene ring and the nitrogen atoms of the triazole contribute moderately negatively to negative regions, suggesting possible participation in weak donor-acceptor interactions. This charge distribution supports the molecule’s dual character as a ligand and an electron-transfer mediator [45, 46].

### Non-covalent interactions (NCI)

**Fig. 9:**
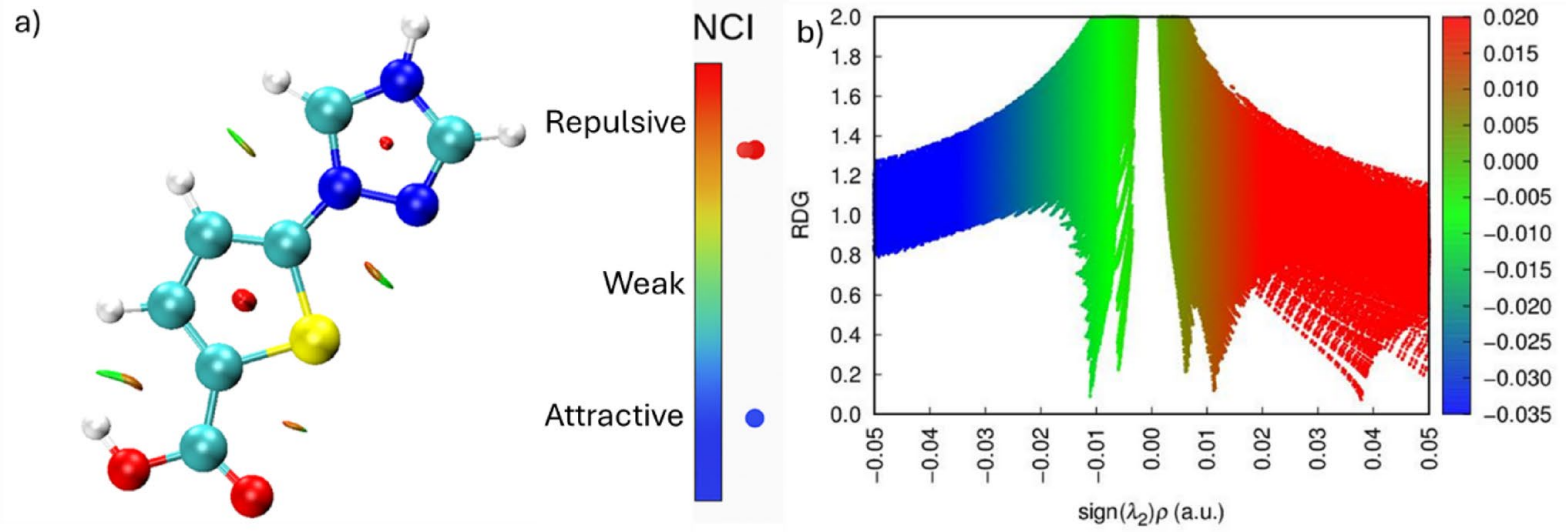
**a** NCI isosurface representation **b** RDG-based of TTCA molecule

Reduced density gradient (RDG) analysis (Fig. 9) was performed to visualize intramolecular weak interactions. In the NCI plot (Fig. 9), the red regions at the center of the rings denote steric repulsion. The broad green iso-surfaces between the thiophene sulfur and the triazole ring indicate dominant van der Waals interactions. Specifically, the interaction between the carboxyl hydrogen (H16) and the thiophene sulfur (S5) appears as a cyan-to-green region, suggesting a moderate interaction driven by electrostatic and dispersive forces, rather than a strong covalent-like hydrogen bond. These weak interactions enhance molecular stability and influence packing behavior. Comparatively, recent computational studies on melamine picric acid and fluoroquinolone π-acceptor complexes have demonstrated that stability in those systems is driven by dominant intermolecular charge-transfer and electrostatic interactions. The NCI analysis of TTCA reveals that its structural integrity is primarily maintained through intramolecular hydrogen bonding and steric stabilization rather than strong intermolecular CT driving forces. This distinction highlights TTCA’s potential as a stable, standalone heteroaromatic scaffold rather than a transient donor–acceptor adduct [47]–[48]. These weak interactions reinforce the planar geometry required for efficient π-conjugation, as evidenced by the continuous green RDG iso-surfaces between the ring systems (Fig. 9).

## Conclusion

In this study, the molecular geometry, electronic configuration, and spectroscopic characteristics of 5-(1 H-1,2,4-triazol-1-yl)-2-thiophenecarboxylic acid (TTCA) were systematically examined through DFT and TD-DFT computations. The optimized structure obtained at the B3LYP/6-311++ G(d, p) level revealed a nearly planar π-conjugated skeleton, stabilized by intramolecular hydrogen bonding. The HOMO-LUMO gap of approximately 3.1 eV, along with a global hardness of 1.55 eV, characterizes TTCA as a chemically hard system with high resistance to intramolecular charge transfer in its ground state. The simulated FT-IR and NMR spectra successfully reproduced the characteristic vibrational and chemical-shift features observed in experimental analogs of the triazole, thiophene, and carboxyl functional groups, validating the computational approach. The UV-Vis analysis revealed strong π→π* transitions around 720 nm, suggesting efficient electronic delocalization and possible use in optoelectronic devices. The DOS and OPDOS profiles further emphasized charge delocalization across the heterocyclic system, while the MEP map identified oxygen and nitrogen atoms as preferred nucleophilic and coordination centers. Non-covalent interaction (NCI) and RDG analyses indicated weak hydrogen-bonding and van der Waals interactions that reinforce molecular planarity and stability. These interactions, combined with a moderate band gap and broad absorption profile, underline TTCA’s suitability for applications in coordination chemistry, sensor technology, and photoactive organic materials. The present work provides a comprehensive theoretical perspective on the structural, electronic, and optoelectronic features of TTCA. While this study established the fundamental properties of the isolated monomer, future investigations should expand into exploring the metal coordination behavior of the N-rich triazole and O-rich carboxyl domains for MOF construction, modeling solid-state packing to predict charge mobility in bulk materials, and designing functionalized derivatives to finely tune the HOMO-LUMO gap for specific photovoltaic or sensing applications. The obtained insights not only advance understanding of heteroaromatic charge-transfer systems but also pave the way for future studies on TTCA-based coordination complexes and functional materials optimized for optoelectronic and sensing purposes.

## Data Availability

The datasets generated and/or analysed during the current study are available from the corresponding author on reasonable request.
